# Oral Chitosan–Tripolyphosphate Nanoparticles Enhance the Metabolic Regulatory Effects of Snow Lotus Polysaccharide in Type 2 Diabetes

**DOI:** 10.3390/pharmaceutics18050561

**Published:** 2026-04-30

**Authors:** Shangyi Huang, Lei Liu, Jiani Li, Hongyang Ren, Huamin Wang, Wantong Zhao, Shuangqing Wang, Guangyao Li, Congshu Dai

**Affiliations:** 1 College of Nursing, Yanbian University, Yanji 133000, China; 2 College of Pharmacy, Yanbian University, Yanji 133000, China; 3 College of Chemistry, Jilin University, Changchun 130012, China; 4 Changchun GeneScience Pharmaceutical Co., Ltd., Changchun 130015, China; 5 Institute of Materia Medica, Chinese Academy of Medical Sciences & Peking Union Medical College, Beijing 100050, China; wangshuangqing@imm.ac.cn; 6 College of Medicine, Yanbian University, Yanji 133000, China

**Keywords:** oral delivery, polysaccharide nanoparticles, metabolic regulation, intestinal permeability, inflammation, cross-linking

## Abstract

**Purpose:** Natural polysaccharides have shown considerable potential in the management of type 2 diabetes mellitus (T2DM) due to their multi-target metabolic regulatory effects. However, their clinical translation is limited by poor oral stability and low intestinal permeability. Snow lotus polysaccharide (SIP), a representative plant-derived polysaccharide, exhibits promising metabolic benefits but suffers from these delivery barriers. This study aimed to develop an oral nanodelivery system to enhance the gastrointestinal stability and intestinal transport of SIP, thereby improving its in vivo efficacy. **Methods:** SIP-loaded chitosan–tripolyphosphate nanoparticles (SIP@CS-TPP) were prepared via ionic crosslinking and characterized in terms of particle size, surface charge, morphology, and structural features. In vitro release behavior under simulated gastrointestinal conditions was evaluated. Ex vivo intestinal permeation was assessed using an isolated intestinal sac model. The metabolic regulatory effects were further investigated in a high-fat diet/streptozotocin-induced T2DM rat model. **Results:** SIP@CS-TPP nanoparticles exhibited a uniform particle size of 188.9 ± 12.8 nm, a surface charge of 28.3 ± 5.1 mV, and good stability after freeze-drying. A pH-responsive and diffusion-controlled release profile was observed. Ex vivo studies demonstrated significantly enhanced intestinal transport, with an approximately 3.7-fold increase in apparent permeability compared with free SIP. In vivo, SIP@CS-TPP improved glycemic control, glucose tolerance, insulin resistance, lipid metabolism, oxidative stress, and inflammatory responses more effectively than free SIP at the same dose. **Conclusions:** The CS-TPP nanodelivery system effectively enhances the oral delivery and metabolic regulatory effects of SIP. This study highlights the potential of a delivery-oriented strategy to improve the in vivo performance of natural polysaccharides and provides a promising approach for their application in metabolic disease management.

## 1. Introduction

Type 2 diabetes mellitus (T2DM) is a chronic metabolic disorder characterized by insulin resistance, impaired glucose homeostasis, and associated abnormalities such as dyslipidemia, oxidative stress, and chronic low-grade inflammation [1,2]. The global prevalence of diabetes continues to increase, with an estimated 589 million adults affected in 2024, and further growth is expected in the coming decades [3]. Although a range of pharmacological therapies is available, long-term management remains challenging. In particular, current treatments often fail to simultaneously regulate multiple metabolic pathways, including glucose metabolism, lipid balance, and inflammatory responses [4].

Natural polysaccharides have attracted increasing attention as potential agents for metabolic diseases due to their favorable safety profiles and multi-target biological activities. Previous studies have shown that plant-derived polysaccharides can improve glycemic control, enhance insulin sensitivity, and regulate metabolic homeostasis. These effects are associated with antioxidant activity, suppression of inflammatory signaling pathways, and modulation of gut microbiota [5,6]. Compared with conventional small-molecule drugs, polysaccharides may provide broader regulatory effects across multiple pathological processes involved in T2DM [7].

Snow lotus polysaccharide (SIP), isolated from *Saussurea involucrata*, is a representative plant-derived polysaccharide with hypoglycemic, antioxidant, and anti-inflammatory properties [8]. Studies on snow lotus extracts and related components have shown that they can suppress pro-inflammatory cytokine production and regulate key signaling pathways, including NF-κB, PI3K, and MAPK, which are closely associated with metabolic inflammation and insulin resistance [9,10,11,12]. In addition, snow lotus and its constituents have been reported to exhibit a broad range of pharmacological activities, such as antioxidant, immunomodulatory, and metabolic regulatory effects [11]. These findings suggest that SIP may improve metabolic dysfunction through coordinated regulation of oxidative stress and inflammatory responses. However, despite these promising properties, SIP’s practical application is severely limited by oral delivery barriers. Its high molecular weight and complex structure cause gastrointestinal degradation and low intestinal permeability, restricting oral absorption and systemic exposure [13]. Existing studies focus on its in vitro or isolated pharmacological effects, with little attention to in vivo delivery barriers and few systematic strategies to boost its efficacy via delivery optimization. Thus, rather than merely verifying SIP’s antidiabetic activity, addressing its delivery limitations to amplify in vivo effects holds greater scientific value and translational potential.

This limitation reflects a common challenge for natural polysaccharides, namely, insufficient delivery efficiency rather than a lack of intrinsic bioactivity. Therefore, improving oral delivery, rather than simply increasing dosage, has become an important strategy to enhance their therapeutic effects [14,15]. Nanodelivery systems provide a feasible approach by protecting bioactive compounds during gastrointestinal transit and facilitating their transport across the intestinal barrier [16,17].

Among various candidate carrier materials, chitosan (CS) is a natural cationic polysaccharide. It has been widely used in the development of oral delivery systems due to its good biocompatibility, biodegradability and intestinal mucoadhesive properties [18,19]. For its core mechanism, the cationic amino groups of CS can reversibly interact with tight junction-related proteins on intestinal epithelial cells, including occludin and zonula occludens-1 (ZO-1) [20]. This interaction transiently opens the intercellular tight junctions of the epithelium and widens the paracellular transport pathway. It thus significantly improves the intestinal permeability of macromolecules and enhances their oral absorption efficiency. This effect is transient and reversible, with no risk of permanent damage to the intestinal barrier [21]. Among CS-based delivery systems, CS-tripolyphosphate (CS-TPP) ionically crosslinked nanoparticles have mild preparation conditions. They can protect active ingredients from degradation in the harsh acidic and enzymatic environment of the gastrointestinal tract, and maximally preserve the bioactivity of the active ingredients. CS-TPP nanoparticles have been proven to enhance the stability and intestinal absorption of insulin, thus improving its bioavailability and sustained hypoglycemic effect [22]. Meanwhile, they have also been confirmed to significantly improve the oral bioavailability and in vivo hypoglycemic activity of natural active ingredients [23]. In addition, this nanosystem has the advantages of tunable particle size and surface potential, as well as easy feasibility for freeze-drying storage and large-scale preparation [24]. Despite this, there are still very few systematic studies on the optimization of SIP oral delivery that simultaneously integrate delivery efficiency improvement, in vivo metabolic improvement validation, and inflammation-related mechanism elucidation.

Based on these considerations, we put forward a clear scientific hypothesis: enhancing the oral delivery efficiency of SIP via CS-TPP nanoparticles can simultaneously protect SIP from gastrointestinal degradation and improve its intestinal epithelial permeability, thus amplifying its in vivo metabolic regulatory and antidiabetic effects. The core aim of this work was to develop and characterize SIP-loaded CS-TPP nanoparticles, and systematically evaluate their physicochemical properties, in vitro release behavior, ex vivo intestinal permeation profile, and therapeutic efficacy in a T2DM animal model. This delivery-oriented strategy is expected to provide a practical and broadly applicable approach to promote the oral translation of natural polysaccharides for metabolic disease management.

## 2. Materials and Methods

### 2.1. Materials

SIP (purity ≥ 80%, lot no. Z259197YQ) was purchased from Yanshen (Tianjin) Technology Co., Ltd. (Tianjin, China). The physicochemical characteristics of SIP were as follows: total polysaccharide content of 82.3 ± 1.2%, protein residue of 2.1 ± 0.3%, ash content of 3.2 ± 0.4%, and moisture content of 5.7 ± 0.5%. The proportion of low-molecular-weight impurities (<1 kDa) was 2.7 ± 0.4%, and the residual ethanol content was 0.28%. The weight-average molecular weight and number-average molecular weight were 3.72 × 10^4^ Da and 1.88 × 10^4^ Da, respectively, with a polydispersity index (PDI) of 1.98. The main active fraction (10–100 kDa) accounted for 91.2 ± 1.5% of the total polysaccharide. The monosaccharide composition (molar ratio) was arabinose:rhamnose:xylose:galactose:glucose:galacturonic acid:mannose = 2.8:2.6:1.0:2.6:2.0:7.6:1.6. Sodium tripolyphosphate (S100099) was obtained from Aladdin Biochemical Technology Co., Ltd. (Shanghai, China). CS with different molecular weights (30 kDa, C850346; 50 kDa, C850347; 100 kDa, C850348; and 150 kDa, C875438) was purchased from Macklin Biochemical Co., Ltd. (Shanghai, China). According to the manufacturer’s certificate of analysis, the molecular weight of these CS samples was determined by gel permeation chromatography using dextran as standard and refractive index detector with 0.2 mol/L sodium sulfate solution (pH 5.0) as mobile phase, column temperature at 35 °C and flow rate of 0.5 mL/min. The degree of deacetylation of all CS samples was ≥85%, which was measured by potentiometric titration according to the supplier’s standard protocol. The high-fat/high-sucrose diet used for animal experiments consisted of 10% lard, 20% sucrose, 2.5% cholesterol, 1% bile salts, and 66.5% standard chow, and was supplied by Fanbo Biotechnology Co., Ltd. (Shanghai, China). Streptozotocin (STZ, WXBC2544V) was obtained from Sigma-Aldrich (St. Louis, MO, USA). Metformin hydrochloride enteric-coated capsules (approval no. H20058567) were purchased from Beijing Shengyong Pharmaceutical Co., Ltd. (Beijing, China). Commercial assay kits for total cholesterol (TC, A111-1-1), triglycerides (TG, F001-1-1), high-density lipoprotein cholesterol (HDL-C, F003-1-1), low-density lipoprotein cholesterol (LDL-C, A113-1-1), and fasting insulin (FINS, H203) were obtained from Nanjing Jiancheng Bioengineering Institute (Nanjing, China).

Forty-two specific pathogen-free (SPF) male Sprague–Dawley (SD) rats, weighing 200 ± 20 g, were purchased from Changchun Yisi Experimental Animal Technology Co., Ltd. (Changchun, China).

### 2.2. Preparation and Formulation Optimization of CS-TPP Nanoparticles

CS-TPP nanoparticles were prepared using the ionic crosslinking method [25,26]. Briefly, CS was dissolved in 1.0% (*v*/*v*) aqueous acetic acid solution, and the pH of the solution was adjusted to the desired value. A TPP aqueous solution at a defined concentration was then added dropwise to the CS solution under magnetic stirring to form a nanoparticle suspension. To optimize the preparation conditions, key parameters including stirring time, CS molecular weight, system pH, CS concentration, TPP concentration, and the CS: TPP mass ratio were systematically investigated with respect to their effects on particle size and PDI.

### 2.3. Encapsulation of SIP and Characterization of Nanoparticles

#### 2.3.1. Preparation of SIP-Loaded CS-TPP Nanoparticles (SIP@CS-TPP)

SIP@CS-TPP were prepared by ionic crosslinking. Briefly, CS (molecular weight approximately 100 kDa) was dissolved in 1.0% (*v*/*v*) aqueous acetic acid to obtain a CS solution with a concentration of 1.0 mg/mL. The pH of the CS solution was gradually adjusted to 5.0 using 1.0 mol/L NaOH solution. SIP was then added to the pH-adjusted CS solution to achieve a final SIP concentration of 1.0 mg/mL, followed by magnetic stirring at 25 °C and 600 rpm until complete dissolution. Separately, TPP was dissolved in deionized water to prepare a 0.5 mg/mL TPP solution. Under continuous magnetic stirring (25 °C, 600 rpm), the TPP solution was slowly added dropwise to the SIP-containing CS solution, while maintaining a CS:TPP mass ratio of 5:1. After completion of TPP addition, the mixture was further stirred for 1.5 h to promote sufficient ionic crosslinking between CS and TPP, resulting in a stable SIP@CS-TPP nanoparticle dispersion. The resulting dispersion was centrifuged at 12,000 rpm for 30 min at 4 °C to separate nanoparticles from unencapsulated free SIP. The supernatant was discarded, and the pellet was collected and resuspended in deionized water to obtain purified SIP@CS-TPP nanoparticles for subsequent characterization and experiments.

#### 2.3.2. Dynamic Light Scattering (DLS) and Zeta Potential Analysis

The hydrodynamic diameter and PDI of blank CS-TPP and SIP@CS-TPP nanoparticles were measured by DLS using a Zetasizer Nano ZS90 (Malvern Panalytical, Malvern, UK). Prior to measurement, nanoparticle dispersions were appropriately diluted with deionized water to a concentration below 0.2 mg/mL to achieve an optimal scattering intensity. Zeta potential was determined by electrophoretic light scattering using the same instrument. All measurements were performed at 25 °C, and each sample was analyzed in triplicate.

#### 2.3.3. Encapsulation Efficiency and Drug Loading

The encapsulation efficiency (EE) of SIP in CS-TPP nanoparticles was determined by measuring the amount of free (unencapsulated) SIP in the supernatant. After nanoparticle formation, the suspension was centrifuged at 12,000 rpm for 30 min at 4 °C. The supernatant was collected, and the concentration of free SIP was determined using the phenol–sulfuric acid method. The concentration of SIP was determined using the phenol–sulfuric acid method, a widely used colorimetric assay for total carbohydrate quantification. In this method, carbohydrates are dehydrated by concentrated sulfuric acid to form furfural derivatives, which react with phenol to produce a colored compound with absorbance measured at 490 nm [27].

The encapsulation efficiency was calculated according to the following equation:

EE (%) = [(W_total_ − W_free_)/W_total_] × 100%

where W_total_ represents the total amount of SIP used in the formulation, and W_free_ represents the amount of free SIP detected in the supernatant.

The drug loading (DL) was calculated as follows:

LC (%) = [(W_total_ − W_free_)/W_nanoparticles_] × 100%

where W_nanoparticles_ represents the total weight of the obtained nanoparticles.

#### 2.3.4. Transmission Electron Microscopy (TEM)

The morphology of the nanoparticles was examined using a transmission electron microscope (JEM-2100, JEOL, Tokyo, Japan). Briefly, a drop of diluted nanoparticle dispersion was deposited onto a carbon-coated copper grid and allowed to adsorb for 1–2 min. Excess liquid was gently removed with filter paper, and the grid was air-dried at room temperature. The samples were negatively stained with 1% (*w*/*v*) phosphotungstic acid for 30 s prior to imaging. TEM images were acquired at an accelerating voltage of 200 kV.

#### 2.3.5. Fourier Transform Infrared (FT-IR) Spectroscopy

FT-IR spectra of SIP, CS, blank CS-TPP nanoparticles, and SIP@CS-TPP nanoparticles were recorded using an FT-IR spectrometer (Nicolet iS50, Thermo Fisher Scientific, Waltham, MA, USA). Freeze-dried samples were analyzed over a spectral range of 4000–400 cm^−1^ with a spectral resolution of 4 cm^−1^. The spectra were used to identify characteristic functional groups and to assess potential interactions between SIP and the CS-TPP nanoparticle matrix.

### 2.4. Freeze-Drying and Storage Stability of SIP@CS-TPP Nanoparticles

Freshly prepared SIP@CS-TPP nanoparticle dispersions were divided into different treatment groups. For samples containing cryoprotectants, mannitol and trehalose were added to the nanoparticle dispersions at final concentrations of 2% (*w*/*v*) each and mixed thoroughly. Samples without cryoprotectants were prepared in parallel as controls. All samples were pre-frozen at −80 °C for 12 h and then subjected to freeze-drying using a lyophilizer (cold trap temperature approximately −50 °C, vacuum < 10 Pa) for 48 h. After lyophilization, the obtained dry powders were sealed and stored at room temperature. For reconstitution, deionized water was added and gently agitated until complete redispersion. The mean particle size and PDI of reconstituted nanoparticles were measured by dynamic light scattering, and nanoparticle morphology was observed using transmission electron microscopy. To evaluate storage stability, freeze-dried samples were stored under dry conditions at room temperature for 1, 2, 3, 4, 5, and 6 months. At predetermined time points, samples were collected, reconstituted, and analyzed for changes in particle size and PDI.

### 2.5. In Vitro Release Study

The in vitro release behavior of SIP@CS-TPP nanoparticles under different pH conditions was evaluated using a dialysis method. Briefly, 2.0 mL of SIP@CS-TPP nanoparticle dispersion was loaded into dialysis bags (molecular weight cutoff: 8–14 kDa), with an SIP concentration of 1.5 mg/mL, corresponding to a total drug amount of 3.0 mg. The dialysis bags were immersed in 50 mL of release medium and incubated at 37 ± 0.5 °C with continuous shaking at 100 rpm. PBS at pH 1.2, 6.8, and 7.4 was used as the release medium to simulate different gastrointestinal environments. Sink conditions were maintained throughout the experiment. At predetermined time points, 1.0 mL of the release medium was withdrawn and immediately replaced with an equal volume of fresh pre-warmed medium to maintain a constant volume and sink conditions. The amount of SIP released into the external medium was quantified using the phenol–sulfuric acid method, and the cumulative release percentage was calculated.

For the simulated gastrointestinal transit experiment, the dialysis bags were sequentially incubated in pH 1.2 medium for 2 h, followed by pH 6.8 medium for 4 h, and then transferred to pH 7.4 medium until the end of the experiment, while all other experimental conditions remained unchanged.

### 2.6. Effect of SIP@CS-TPP on Intestinal Permeability

#### 2.6.1. Ex Vivo Intestinal Permeation Study

An ex vivo intestinal sac model was employed to evaluate the intestinal permeability of free SIP and SIP@CS-TPP nanoparticles. SD rats were fasted overnight and placed in a sealed anesthesia chamber (volume: 1000 mL, adapted for 200–240 g SD rats) prefilled with 5% isoflurane (balanced with 100% medical oxygen at a flow rate of 1.5 L/min). The chamber was maintained in a darkened environment to reduce environmental stress, and rats were continuously monitored for behavioral responses. Anesthesia was confirmed by the absence of pedal reflex and corneal reflex within 3–5 min of exposure, after which rats were sacrificed by cervical dislocation. The small intestine was immediately excised, and segments approximately 4 cm in length were collected. Both ends of each segment were ligated to form closed intestinal sacs with an effective permeation area of approximately 7.85 cm^2^. Each intestinal sac was filled with 0.2 mL of test solution, in which the initial SIP concentration was 1.0 mg/mL for both free SIP and SIP@CS-TPP. The sacs were then placed in containers containing 5.0 mL of pH 7.4 PBS and incubated at 37 °C with gentle shaking at 50 rpm. Sink conditions were maintained throughout the experiment. At 0.5, 1, 2, 3, 4, 5, and 6 h, 0.5 mL of the external medium was collected and immediately replaced with an equal volume of fresh, pre-warmed PBS. The amount of SIP permeated into the external medium was determined using the phenol-sulfuric acid method, and the cumulative permeation amount was calculated. The apparent permeability coefficient (Papp) was calculated according to the following Equation (1) [28]:

Papp = (d_Q_/d_t_)/(A·C_0_), (1)

where d_Q_/d_t_ represents the steady-state slope of the cumulative permeated amount of SIP over time, A is the effective permeation area (7.85 cm^2^), Q represents the cumulative percentage of SIP released at time t, t denotes the release time, and C_0_ is the initial SIP concentration inside the intestinal sac (1.0 mg/mL).

#### 2.6.2. Ex Vivo Intestinal Fluorescence Imaging

To visually assess the effect of CS-TPP nanoparticles on trans-epithelial transport, the near-infrared fluorescent dye IR820 was used as a tracer molecule to replace SIP for ex vivo intestinal fluorescence imaging. The preparation procedure for IR820@CS-TPP nanoparticles was identical to that of SIP@CS-TPP, except that SIP was replaced with an equivalent mass of IR820. Briefly, IR820 was added to the CS solution at a final concentration of 1.0 mg/mL and stirred at 25 °C and 600 rpm until homogeneous, followed by dropwise addition of the TPP solution to form IR820@CS-TPP nanoparticles. The ex vivo intestinal sac model was established as described in Section 2.6.1. After completion of the permeation experiment, the intestinal tissues were gently rinsed with cold PBS to remove residual fluorescence on the surface, followed by embedding and cryosectioning. Tissue sections were stained with DAPI to label cell nuclei and subsequently observed using confocal laser scanning microscopy (CLSM, Biotek, Winooski, VT, USA) to visualize the distribution of IR820 within the intestinal tissue.

### 2.7. Animal Experiments

The high-fat/high-sucrose diet combined with STZ was used to establish a T2DM animal model [29,30,31]. The animal experiments were approved by the Animal Ethics Committee of Yanbian University (Approval No. YD20241211007). Experimental animals were allowed to acclimatize for one week under standard laboratory conditions and were then fed a high-fat diet for 4 weeks. Subsequently, diabetes was induced by a single intraperitoneal injection of STZ (35 mg/kg), freshly dissolved in 0.1 mol/L citrate buffer (pH 4.5). Fasting blood glucose (FBG) levels were measured after STZ administration, and animals with FBG ≥ 11.1 mmol/L were considered successfully diabetic. Diabetic animals were randomly divided into the model group, SIP group, SIP@CS-TPP group, and positive control group, 6 rats in each group. An additional normal control group was included. Animals in the SIP and SIP@CS-TPP groups received oral administration by gavage. The dose of SIP was 100 mg/kg, administered once daily for 8 consecutive weeks. The SIP@CS-TPP group received an equivalent SIP dose of 100 mg/kg/day, calculated based on SIP content. Animals in the normal control and model groups were administered an equal volume of normal saline. Metformin hydrochloride was used as the positive control. It was administered orally at a dose of 150 mg/kg/day during the experimental period.

Throughout the experimental period, all animals were allowed free access to food and water. Body weight and fasting blood glucose levels were recorded at regular intervals. During the treatment period, oral glucose tolerance tests (OGTT) were performed at predetermined time points. At the end of the experiment, rats were fasted overnight and subjected to 5% isoflurane inhalation euthanasia using a dedicated veterinary anesthesia system. Rats were placed individually in a well-ventilated, opaque anesthesia chamber (volume: 800 mL) to avoid social stress from conspecifics. 5% isoflurane (carried by 100% oxygen at 2 L/min) was introduced into the chamber to induce rapid unconsciousness, with continuous monitoring of respiratory rate and behavioral responses. Unconsciousness was verified by the loss of righting reflex, corneal reflex, and voluntary breathing within 4–6 min. To ensure definitive death, 5% isoflurane exposure was continued for an additional 5 min after the cessation of spontaneous breathing. Death was ultimately confirmed by three independent criteria: absence of respiratory movement for ≥5 min, no palpable heartbeat at the thoracic region, and fixed, dilated pupils unresponsive to light stimulation. Blood samples and relevant tissues were collected for biochemical analysis, assessment of oxidative stress markers, measurement of inflammatory cytokine levels. All animal experiments were conducted in strict accordance with institutional guidelines and ethical regulations for the care and use of laboratory animals.

### 2.8. Statistical Analysis

Statistical analyses were performed using GraphPad Prism 9.5 software. All data are presented as mean ± SD, and each experiment was conducted with at least three independent replicates. Comparisons between two groups were performed using a two-tailed unpaired Student’s *t*-test, while comparisons among multiple groups were analyzed by one-way analysis of variance (ANOVA) followed by appropriate post hoc multiple comparison tests. A value of *p* < 0.05 was considered statistically significant, and statistical significance is indicated as * *p* < 0.05, ** *p* < 0.01, and *** *p* < 0.001.

## 3. Results

### 3.1. Optimization of Formulation Parameters and Characterization of CS-TPP Nanoparticles

To obtain CS-TPP nanoparticles with uniform particle size and good dispersibility, key formulation and process parameters were systematically optimized, including stirring time, CS molecular weight, system pH, CS concentration, TPP concentration, and the CS: TPP mass ratio. The effects of these parameters on particle size and PDI were evaluated.

#### 3.1.1. Effect of Stirring Time

**Figure 1:**
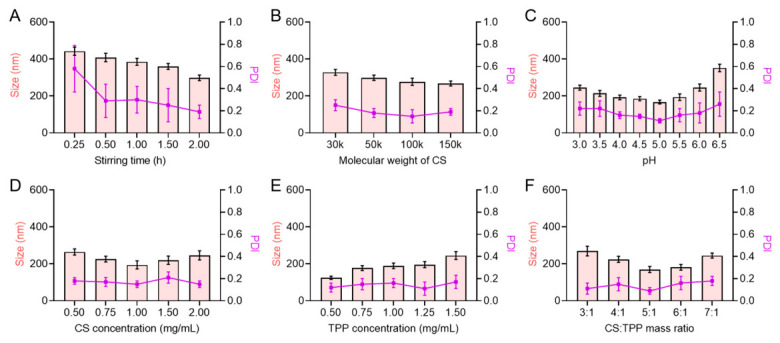
Optimization of formulation parameters for CS/TPP nanoparticles, n = 3. Effects of (**A**) stirring time, (**B**) molecular weight of CS, (**C**) pH value, (**D**) CS concentration, (**E**) TPP concentration, and (**F**) CS: TPP mass ratio on particle size and PDI of CS-TPP nanoparticles.

Stirring time is a critical kinetic factor governing the extent of ionic crosslinking and structural homogenization during CS-TPP nanoparticle formation. As the stirring time was extended from 0.25 h to 2.0 h, the mean particle size of CS-TPP nanoparticles gradually decreased from approximately 440 nm to 290 nm (Figure 1A, Table S1), accompanied by a pronounced reduction in PDI. At shorter stirring times, ionic crosslinking between CS and TPP was incomplete, resulting in larger particle sizes and broader size distributions. Prolonged stirring facilitated sufficient electrostatic interaction and densification of the crosslinked structure, leading to smaller and more uniformly distributed nanoparticles. However, when the stirring time exceeded 1.5 h, the decrease in particle size became marginal, indicating that the nanoparticle system had reached a relatively stable state.

#### 3.1.2. Effect of CS Molecular Weight

The molecular weight of CS determines chain length and entanglement behavior, which directly influence the compactness and stability of ionically crosslinked nanoparticles. All CS samples with different molecular weights used in this study had an identical degree of deacetylation (≥85%), eliminating the interference of this parameter on the crosslinking behavior. As shown in Figure 1B, increasing the molecular weight of CS from 30 kDa to 150 kDa resulted in a slight decrease in particle size. Relatively small and stable nanoparticles with a low PDI were obtained at approximately 100 kDa. Low-molecular-weight CS possesses shorter polymer chains, which tend to form relatively loose crosslinked networks. In contrast, excessively high molecular weight may enhance chain entanglement, limiting further structural compaction. CS with a moderate molecular weight was therefore favorable for forming structurally stable nanoparticles with good dispersibility.

#### 3.1.3. Effect of System pH

System pH critically affects the protonation state of CS amino groups and thus modulates electrostatic interactions with TPP. The consistent degree of deacetylation of CS used in this study ensured the comparability of charge response under different pH conditions. As shown in Figure 1C, within the pH range of 3.0–5.0, the particle size gradually decreased and reached a minimum at approximately pH 5.0. When the pH was further increased to 6.5, a marked increase in particle size and PDI was observed. This behavior is closely associated with the protonation degree of CS. At lower pH values, CS is highly protonated, which promotes electrostatic interaction with TPP. In contrast, reduced protonation at higher pH weakens ionic crosslinking efficiency, leading to larger particle sizes and broader size distributions.

#### 3.1.4. Effect of CS Concentration

CS concentration controls polymer availability and solution viscosity, thereby influencing nanoparticle nucleation and growth. As illustrated in Figure 1D, increasing the CS concentration from 0.5 mg/mL to 1.0 mg/mL resulted in a gradual decrease in nanoparticle size, reaching the minimum value at 1.0 mg/mL. Further increasing the CS concentration to 2.0 mg/mL caused a slight increase in particle size. An appropriate CS concentration favors the formation of a compact crosslinked network, whereas excessive concentration may increase system viscosity and hinder homogeneous nanoparticle formation, thereby compromising particle size control.

#### 3.1.5. Effect of TPP Concentration

TPP concentration determines the density of crosslinking sites and the degree of ionic network formation. As shown in Figure 1E, increasing the concentration of TPP led to a gradual increase in nanoparticle size. At lower TPP concentrations, insufficient crosslinking sites resulted in relatively loose nanoparticle structures. Conversely, excessive TPP may induce over-crosslinking, causing structural expansion and increased particle size.

#### 3.1.6. Effect of CS: TPP Mass Ratio

The CS: TPP mass ratio governs the balance between positive and negative charges, which is essential for forming structurally stable nanoparticles. As shown in Figure 1F, nanoparticle size initially decreased and then increased with varying CS: TPP mass ratios. Among the tested conditions, a CS: TPP ratio of 5:1 yielded nanoparticles with smaller particle size and lower PDI, indicating that an appropriate charge balance is critical for achieving uniform and stable CS-TPP nanoparticles.

Overall, the optimization results showed that favorable CS-TPP nanoparticles were obtained under moderately acidic conditions and balanced formulation parameters. Specifically, relatively small particle size and low PDI were achieved at approximately 100 kDa CS molecular weight, pH 5.0, and a CS concentration of 1.0 mg/mL. Lower TPP concentration was favorable for maintaining smaller particle size, while a CS:TPP mass ratio of 5:1 produced nanoparticles with improved uniformity and stability. In addition, prolonging the stirring time reduced particle size and PDI, although the effect became marginal after 1.5 h, suggesting that the nanoparticle system had approached a relatively stable state.

### 3.2. Encapsulation and Characterization of SIP in CS-TPP Nanoparticles

Under the optimized preparation conditions, SIP was incorporated into the CS-TPP nanoparticle system, and its encapsulation efficiency as well as the effects of loading on the physicochemical properties and structural characteristics of the nanoparticles were systematically evaluated.

#### 3.2.1. EE and DL of Snow Lotus Polysaccharide

**Figure 2:**
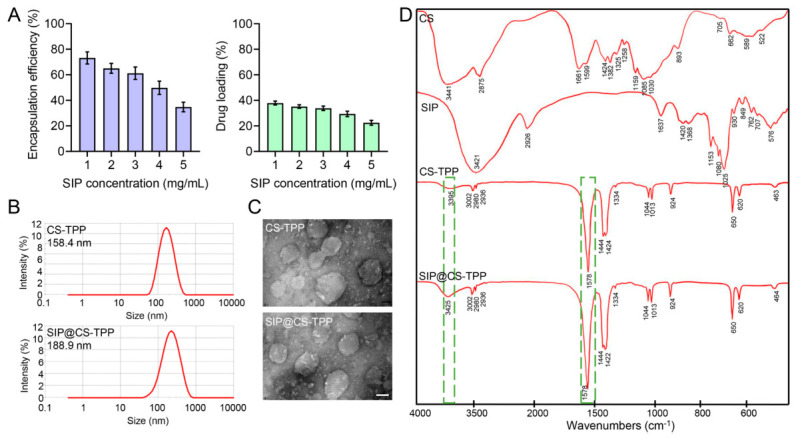
Encapsulation and physicochemical characterization of SIP@CS-TPP nanoparticles. (**A**) Encapsulation efficiency of SIP at different initial SIP concentrations, n = 3. (**B**) Particle size distribution of blank CS-TPP nanoparticles and SIP@CS-TPP nanoparticles measured by DLS. (**C**) TEM images showing the morphology of CS-TPP and SIP@CS-TPP nanoparticles (scale bar = 100 nm). (**D**) FT-IR spectra of CS, SIP, CS-TPP, and SIP@CS-TPP nanoparticles.

EE reflects the loading capacity of the nanoparticle system and is a key parameter for subsequent biological evaluation. Increasing the initial SIP feeding concentration from 1 mg/mL to 5 mg/mL resulted in a gradual decrease in encapsulation efficiency (Figure 2A, Table S2). At relatively low SIP concentrations (1–2 mg/mL), CS-TPP nanoparticles achieved high encapsulation efficiencies exceeding 60%. However, further increases in SIP concentration led to a marked reduction in encapsulation efficiency. This trend is likely attributable to the limited internal space and available binding sites within the nanoparticle matrix, whereby excessive polysaccharides could not be fully accommodated and remained unencapsulated.

In addition to encapsulation efficiency, the DL of SIP in CS-TPP nanoparticles was also calculated. As the initial SIP concentration increased from 1 to 5 mg/mL, the DL decreased from 37.90 ± 1.52% to 22.46 ± 1.88%, showing a trend consistent with that of EE. This trend may be attributed to the limited loading capacity of the CS-TPP matrix. As the amount of SIP increased, the available cross-linking sites and internal space within the nanoparticles gradually became saturated, resulting in a higher proportion of unencapsulated SIP in the supernatant. Consequently, both EE and DL decreased at higher initial SIP concentrations.

#### 3.2.2. Changes in Particle Size and Zeta Potential

Particle size and surface charge are critical determinants of nanoparticle stability and oral delivery performance. DLS analysis (Figure 2B) showed that the mean particle size increased from 158.4 ± 11.6 nm for blank CS-TPP nanoparticles to 188.9 ± 12.8 nm after SIP loading, indicating a moderate size enlargement following polysaccharide incorporation. The particle size of SIP@CS-TPP nanoparticles was approximately 150–200 nm, which falls within a suitable range for oral delivery. Previous studies have suggested that nanoparticles within this size range can facilitate intestinal uptake and improve mucosal transport efficiency [32,33,34]. Zeta potential measurements revealed that blank CS-TPP nanoparticles exhibited a pronounced positive surface charge of 31.9 ± 4.2 mV. After SIP loading, the zeta potential of SIP@CS-TPP nanoparticles slightly decreased to 28.3 ± 5.1 mV. This reduction may be attributed to partial shielding of the positively charged CS surface by hydroxyl groups or weakly anionic moieties present in SIP. Despite this decrease, the nanoparticles maintained a relatively high positive surface charge, which is favorable for colloidal stability in aqueous media and interaction with the intestinal epithelium.

#### 3.2.3. Morphological Characterization of Nanoparticles

The morphological characteristics of the nanoparticles were examined by TEM. As shown in Figure 2C, blank CS-TPP nanoparticles exhibited a nearly spherical morphology with a relatively uniform size distribution. Following SIP encapsulation, SIP@CS-TPP nanoparticles retained their intact spherical structure, with no obvious collapse or aggregation observed. These results indicate that the polysaccharide loading process did not compromise the structural integrity of the nanoparticles.

#### 3.2.4. FT-IR Spectroscopy Analysis

FT-IR spectroscopy was used to characterize the molecular interactions among the samples. As shown in Figure 2D, CS exhibited characteristic stretching vibration bands of –OH and –NH groups around 3400 cm^−1^, with amide I and amide II bands at 1661.4 cm^−1^ and 1598.5 cm^−1^, respectively. After complexation with TPP, the amide I and II bands of CS shifted obviously and merged into a characteristic peak at 1577 cm^−1^, accompanied by distinct absorption bands of phosphate groups in the 1200–900 cm^−1^ region. These changes indicate electrostatic interactions between CS and TPP, which may be attributed to ionic crosslinking. In the FT-IR spectrum of SIP@CS-TPP, no obvious shift was observed in the amide peaks, and the characteristic peaks of SIP remained well preserved without new bands or significant displacements. These results suggest that SIP was mainly loaded into the nanoparticles through physical encapsulation, without forming new covalent bonds with the carrier.

Overall, SIP was successfully encapsulated into CS-TPP nanoparticles, and the drug loading process did not significantly change the particle size distribution, surface charge, or structural integrity of the nanoparticles. These favorable physicochemical characteristics support the suitability of SIP@CS-TPP nanoparticles as an oral delivery system for polysaccharide-based bioactive compounds.

### 3.3. Evaluation of Freeze-Drying and Long-Term Storage Stability of SIP@CS-TPP Nanoparticles

To evaluate the stability of SIP@CS-TPP nanoparticles during solidification and long-term storage, the freeze-drying behavior and post-lyophilization physicochemical properties of the nanoparticles were systematically investigated.

#### 3.3.1. Effect of Cryoprotectants on Nanoparticle Structure

**Figure 3:**
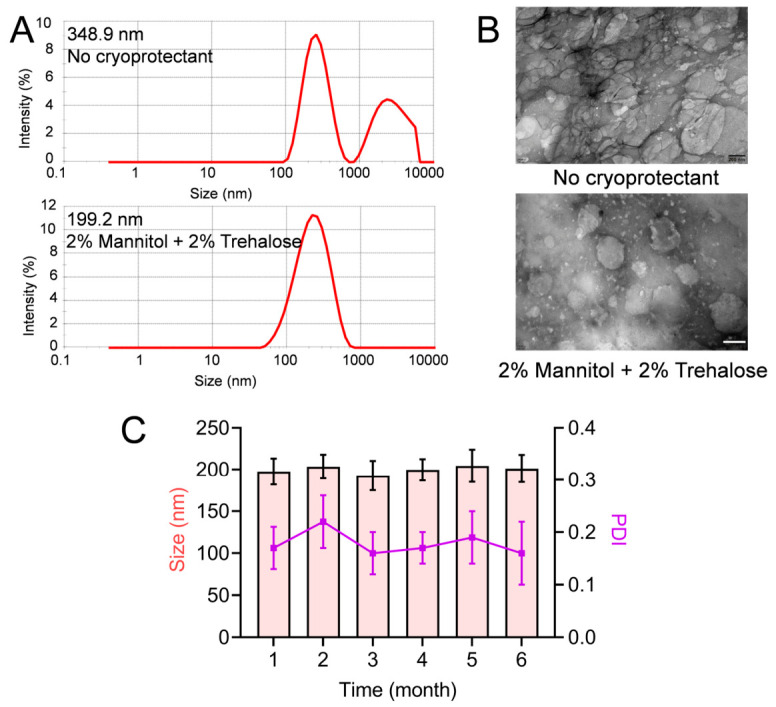
Freeze-drying behavior and storage stability of SIP@CS-TPP nanoparticles. (**A**) Particle size distribution of SIP@CS-TPP nanoparticles after freeze-drying with or without cryoprotectants. (**B**) TEM images of SIP@CS-TPP nanoparticles after freeze-drying without cryoprotectant and with 2% (*w*/*v*) mannitol plus 2% (*w*/*v*) trehalose (scale bar = 100 nm). (**C**) Changes in particle size and PDI of freeze-dried SIP@CS-TPP nanoparticles during storage at room temperature for up to 6 months, n = 3.

SIP@CS-TPP nanoparticles freeze-dried without cryoprotectants exhibited a marked increase in mean particle size to 348.9 ± 28.4 nm after reconstitution (Figure 3A), accompanied by the appearance of a pronounced multimodal size distribution, indicating severe nanoparticle aggregation during the freeze-drying process. Corresponding TEM images (Figure 3B) revealed substantial structural collapse and fusion of nanoparticles in the absence of cryoprotectants, with blurred particle boundaries, further confirming irreversible damage to the nanostructure induced by freeze-drying. In contrast, when 2% (*w*/*v*) mannitol and 2% (*w*/*v*) trehalose were added as cryoprotectants, the reconstituted SIP@CS-TPP nanoparticles maintained a mean particle size of approximately 199.2 ± 13.5 nm, with a unimodal size distribution and no obvious formation of large aggregates. TEM observations demonstrated that nanoparticles preserved a relatively intact spherical morphology and good dispersibility in the presence of cryoprotectants. These results indicate that appropriate cryoprotectants can effectively mitigate nanoparticle damage caused by ice crystal formation and dehydration during freeze-drying, thereby maintaining colloidal stability.

#### 3.3.2. Long-Term Storage Stability After Freeze-Drying

The long-term storage stability of freeze-dried SIP@CS-TPP nanoparticles containing cryoprotectants was further evaluated. As shown in Figure 3C and Table S3, during storage under dry conditions at room temperature for 1–6 months, the mean particle size of the nanoparticles remained within the range of 190–210 nm, while the PDI consistently remained below 0.3, with no significant changes observed over time. These findings demonstrate that SIP@CS-TPP nanoparticles processed by freeze-drying in the presence of suitable cryoprotectants exhibit good physical stability during long-term storage, without apparent aggregation or structural degradation.

Overall, the incorporation of mannitol and trehalose as cryoprotectants significantly enhanced the structural integrity and dispersion stability of SIP@CS-TPP nanoparticles during freeze-drying and prolonged storage, providing an important basis for their further development as an oral nanomedicine formulation.

### 3.4. In Vitro Release Behavior

**Figure 4:**
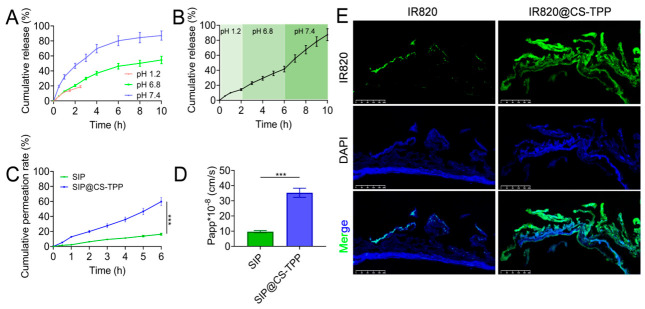
In vitro release behavior and intestinal permeability of SIP@CS-TPP nanoparticles. (**A**) Cumulative release profiles of SIP@CS-TPP nanoparticles under different pH conditions (pH 1.2, 6.8, and 7.4), n = 3. (**B**) Simulated gastrointestinal sequential release profile of SIP@CS-TPP nanoparticles, n = 3. (**C**) Cumulative permeation of free SIP and SIP@CS-TPP across isolated rat small intestine over 6 h, n = 3, *** *p* < 0.001. (**D**) Papp of SIP and SIP@CS-TPP, n = 3, *** *p* < 0.001. (**E**) Representative CLSM images of ex vivo intestinal tissues after treatment with IR820 or IR820@CS-TPP; nuclei were stained with DAPI (scale bar = 200 μm).

To simulate the release characteristics of SIP@CS-TPP nanoparticles in the gastrointestinal environment, their in vitro release behavior was systematically evaluated under pH 1.2, pH 6.8, and pH 7.4 conditions (Figure 4A, Tables S4 and S5). Under simulated gastric conditions (pH 1.2), the cumulative release of SIP from SIP@CS-TPP nanoparticles remained below 21.38 ± 1.62% within 4 h, exhibiting a pronounced sustained-release profile. This result indicates that the nanoparticles possess good structural stability in a strongly acidic environment and can effectively prevent premature polysaccharide release in the stomach. At pH 6.8, the release rate of SIP increased markedly, with a cumulative release of approximately 54.61 ± 4.93% observed within 10 h, showing a sustained and gradual release pattern. Under pH 7.4 conditions, SIP release was further accelerated, reaching 87.21 ± 6.12% within 10 h, indicating a more pronounced release behavior in a neutral environment.

Further evaluation using a simulated sequential “stomach–intestine” transition model (Figure 4B, Table S6) revealed that SIP@CS-TPP nanoparticles exhibited slow release during the initial 2 h at pH 1.2, followed by a progressive increase in release rate after transfer to pH 6.8 and pH 7.4 media. Overall, the nanoparticles displayed a stage-dependent release profile that closely mimics gastrointestinal transit, demonstrating clear pH-responsive release behavior that is favorable for achieving temporally controlled release during oral delivery.

To further elucidate the release kinetics of SIP@CS-TPP nanoparticles under different pH conditions, the release profiles were fitted using Zero-order, First-order, Higuchi, and Korsmeyer–Peppas, and Peppas–Sahlin models (Table 1). The Higuchi model showed the highest goodness of fit across all pH conditions (R^2^ = 0.954–0.992), indicating a diffusion-dominated release profile. The Korsmeyer-Peppas model yielded release exponent (n) values of 0.72–0.88, suggesting an anomalous non-Fickian transport mechanism combining Fickian diffusion and polymer chain relaxation. The Peppas–Sahlin model was further applied to quantify the relative contribution of each mechanism, and the results showed that Fickian diffusion contributed 82.6–86.5% of the total drug release at all tested pH values, which was markedly higher than that of polymer relaxation. These findings collectively confirmed that the release of SIP from CS-TPP nanoparticles was predominantly governed by a diffusion-controlled mechanism. Furthermore, the contribution of Fickian diffusion to SIP release increased gradually as the pH of the release medium rose from 1.2 to 7.4, indicating that the swelling of nanoparticles mediated by elevated pH further enhanced the diffusion-dominated release behavior.

### 3.5. Intestinal Permeation Behavior of SIP@CS-TPP

An ex vivo small intestine permeation model was employed to systematically evaluate the effect of the CS-TPP nanocarrier on the intestinal permeability of snow lotus polysaccharide. Using an initial SIP concentration of 1.0 mg/mL, the permeation behaviors of free SIP and SIP@CS-TPP were compared under identical experimental conditions. The cumulative amount of free SIP permeated into the external medium increased slowly over the 6 h period (Figure 4C, Table S7), reaching only 16.33 ± 1.25% at 6 h. In contrast, the SIP@CS-TPP group exhibited significantly higher permeation at all time points, with the cumulative permeated amount increasing in an approximately linear manner and reaching 59.83 ± 5.31% at 6 h, which was markedly higher than that of free SIP. Notably, at early time points (0.5–1 h), SIP@CS-TPP already demonstrated a significantly higher permeation amount compared with free SIP, indicating that the nanodelivery system effectively promoted the initial trans-intestinal transport of SIP. With prolonged incubation, the difference between the two groups further widened, suggesting that CS-TPP nanoparticles continuously enhanced SIP permeation throughout the entire experimental period. Based on the slope of the cumulative permeation–time profiles during the steady-state phase, the Papp was calculated (Figure 4D, Table S8). The Papp value of free SIP was (9.63 ± 0.74) × 10^−8^ cm/s, whereas SIP@CS-TPP exhibited a significantly increased Papp of (3.53 ± 0.31) × 10^−7^ cm/s, representing an approximately 3.7-fold enhancement compared with free SIP.

Ex vivo intestinal fluorescence imaging further supported the permeation results (Figure 4E). In intestinal sections from the IR820 group, only weak and scattered red fluorescence signals were observed, predominantly confined to the luminal surface, with minimal penetration into deeper mucosal layers, indicating limited trans-epithelial transport of free IR820. In contrast, IR820@CS-TPP-treated tissues displayed markedly enhanced fluorescence intensity. Red fluorescence was not only present on the luminal surface but also clearly extended into the intestinal villi and mucosal layers, showing broader spatial overlap with DAPI-stained nuclei.

Collectively, these results demonstrate that the CS-TPP nanodelivery system significantly enhances the intestinal permeability and tissue distribution of snow lotus polysaccharide, providing a critical delivery basis for its improved antidiabetic and metabolic regulatory effects in vivo.

### 3.6. Effects of SIP@CS-TPP on Overall Metabolic Status in T2DM Rats

**Figure 5:**
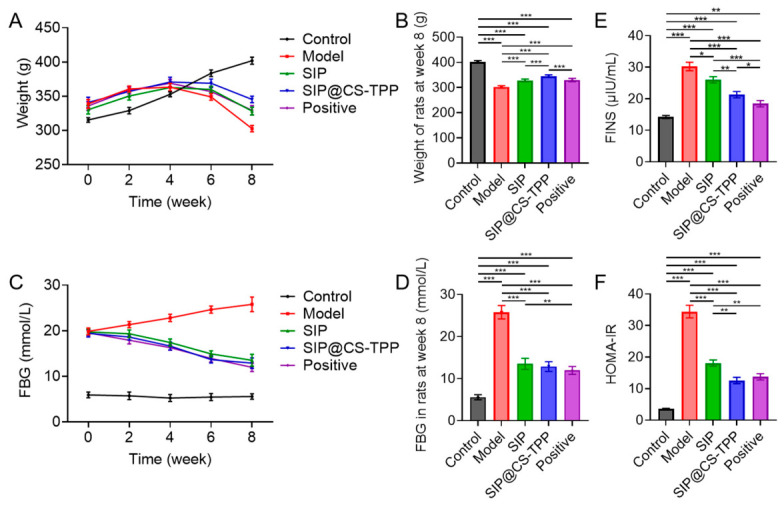
Effects of SIP@CS-TPP on body weight, glycemic control, and insulin resistance in T2DM rats. (**A**) Changes in body weight during the 8-week treatment period, n = 6. (**B**) Body weight at week 8, n = 6, *** *p* < 0.001. (**C**) FBG levels during the treatment period, n = 6. (**D**) FBG levels at week 8, n = 6, ** *p* < 0.01, *** *p* < 0.001. (**E**) FINS levels, n = 6, * *p* < 0.05, ** *p* < 0.01, *** *p* < 0.001. (**F**) HOMA-IR, n = 6, ** *p* < 0.01, *** *p* < 0.001.

Body weight changes and FBG levels are key indicators reflecting overall metabolic status and disease progression in diabetic rats. Throughout the 8-week treatment period, animals in the model group exhibited a progressive decrease in body weight (Figure 5A,B, Table S9), whereas body weight in the normal control group increased steadily, indicating pronounced energy metabolism dysregulation in diabetic rats. Compared with the model group, SIP treatment partially attenuated body weight loss. Notably, rats treated with SIP@CS-TPP showed a more pronounced preservation of body weight, with weight curves consistently higher than those of the free SIP group and gradually approaching the level of the positive control group. By the end of week 8, body weight in the SIP@CS-TPP group was significantly higher than that in the model group.

Dynamic monitoring of FBG revealed a continuous increase in blood glucose levels in the model group over time. Both SIP and SIP@CS-TPP treatments significantly suppressed this upward trend (Figure 5C,D, Table S10). Among them, SIP@CS-TPP exhibited a more stable and pronounced glucose-lowering effect throughout the treatment period, with FBG levels at week 8 significantly lower than those observed in both the model group and the free SIP group.

Further assessment of insulin-related parameters showed that diabetic rats in the model group displayed markedly elevated FINS levels accompanied by a significant increase in the homeostasis model assessment of insulin resistance (HOMA-IR) (Figure 5E,F, Table S11), confirming the presence of insulin resistance. In contrast, SIP@CS-TPP treatment significantly reduced both FINS levels and HOMA-IR values, with improvements exceeding those achieved by free SIP.

Collectively, these findings demonstrate that SIP@CS-TPP markedly improves the overall metabolic status of diabetic rats, effectively reduces hyperglycemia, and alleviates insulin resistance. Importantly, its in vivo glucose-lowering efficacy is superior to that of free snow lotus polysaccharide.

### 3.7. Effects of SIP@CS-TPP on Glucose Tolerance

**Figure 6:**
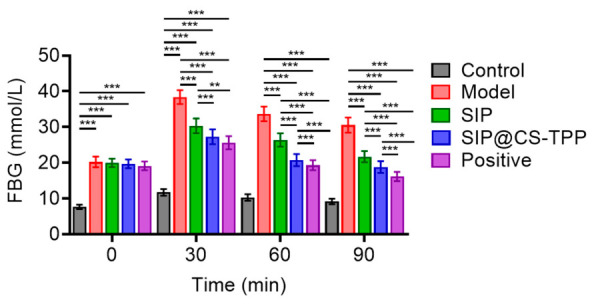
Effects of SIP@CS-TPP on oral glucose tolerance in T2DM rats. Blood glucose levels measured during OGTT at 0, 30, 60, and 90 min in control, model, SIP, SIP@CS-TPP, and positive control groups, n = 6, ** *p* < 0.01, *** *p* < 0.001.

Glucose tolerance reflects the ability of the organism to regulate blood glucose levels under glucose challenge and is a critical indicator of integrated glucose metabolic function. OGTT results showed that diabetic rats in the model group exhibited a rapid increase in blood glucose levels following glucose administration (Figure 6, Table S12), which remained at elevated levels for up to 90 min, indicating severely impaired glucose clearance capacity. In contrast, SIP treatment moderately reduced post-load blood glucose levels. Notably, SIP@CS-TPP treatment resulted in significantly lower blood glucose levels at all measured time points compared with the model group, with a more pronounced downward trend, indicating faster glucose clearance and improved tolerance recovery. The blood glucose levels in the SIP@CS-TPP group at 30, 60, and 90 min were consistently lower than those observed in the free SIP group. These findings demonstrate that SIP@CS-TPP markedly improves glucose tolerance in diabetic rats, highlighting its clear advantage in regulating postprandial glucose metabolism.

### 3.8. Effects of SIP@CS-TPP on Dyslipidemia

**Figure 7:**
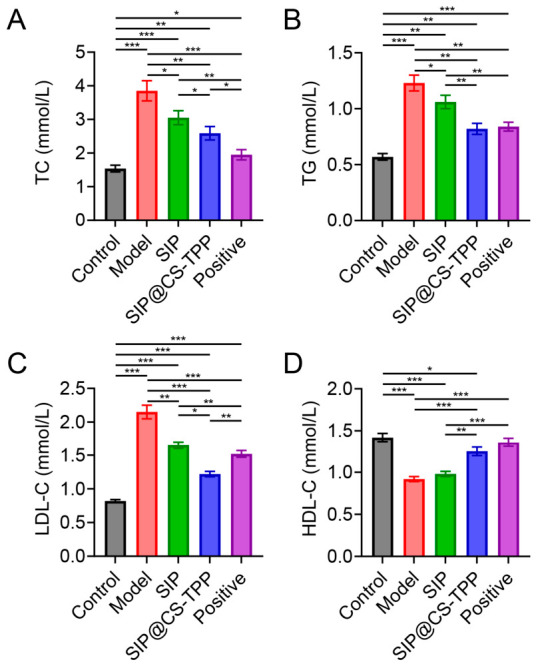
Effects of SIP@CS-TPP on serum lipid metabolism in T2DM rats. (**A**) TC, (**B**) TG, (**C**) LDL-C, and (**D**) HDL-C levels in different treatment groups, n = 6, * *p* < 0.05, ** *p* < 0.01, *** *p* < 0.001.

Dyslipidemia commonly accompanies diabetes and contributes to aggravated insulin resistance and metabolic complications [35]. Serum biochemical analysis revealed that (Figure 7, Table S13), compared with the normal control group, diabetic rats in the model group exhibited significantly elevated levels of TC, TG, and LDL-C, along with a marked reduction in HDL-C, indicating a typical lipid metabolic disorder. SIP treatment partially improved lipid profiles, as evidenced by moderate reductions in TC, TG, and LDL-C levels. In contrast, SIP@CS-TPP treatment produced more pronounced improvements, with TC, TG, and LDL-C levels significantly lower than those in both the model and free SIP groups. Meanwhile, HDL-C levels were significantly increased in the SIP@CS-TPP group, approaching those observed in the positive control group. These results indicate that SIP@CS-TPP effectively ameliorates diabetes-associated dyslipidemia and exhibits potential advantages in correcting the coordinated imbalance between glucose and lipid metabolism.

### 3.9. Effects of SIP@CS-TPP on Oxidative Stress

**Figure 8:**
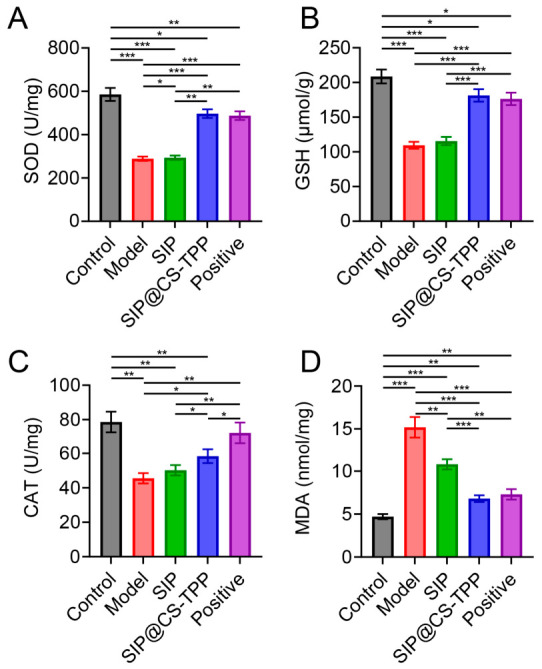
Effects of SIP@CS-TPP on oxidative stress markers in liver tissues of T2DM rats. (**A**) SOD, (**B**) GSH, (**C**) CAT, and (**D**) MDA levels in liver tissues, n = 6, * *p* < 0.05, ** *p* < 0.01, *** *p* < 0.001.

Oxidative stress plays a critical role in the initiation and progression of diabetes and represents a major mechanism underlying tissue injury and metabolic dysfunction [36,37,38]. Glutathione (GSH) is essential for maintaining intracellular redox balance and cellular function, serving as a key antioxidant and detoxifying agent [39,40,41]. Assessment of antioxidant-related markers in liver tissue showed that diabetic rats in the model group exhibited significantly reduced activities of superoxide dismutase (SOD), GSH, and catalase (CAT), accompanied by a marked increase in malondialdehyde (MDA) levels (Figure 8, Table S13), indicating severe oxidative stress damage under diabetic conditions. In contrast, SIP@CS-TPP treatment significantly elevated SOD, GSH, and CAT levels while markedly reducing MDA content, with improvements superior to those achieved by free SIP. These results demonstrate that SIP@CS-TPP effectively enhances endogenous antioxidant defense capacity and alleviates diabetes-associated oxidative stress, providing an important mechanistic basis for its overall metabolic protective effects.

### 3.10. SIP@CS-TPP Attenuates Inflammatory Responses

**Figure 9:**
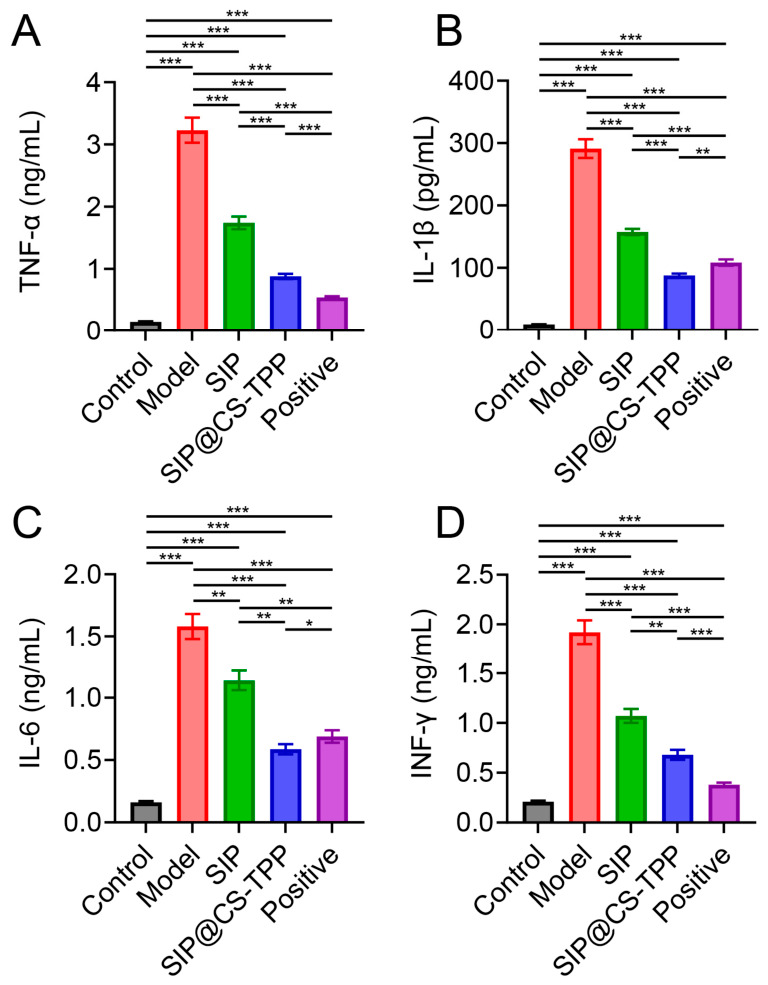
Effects of SIP@CS-TPP on inflammatory cytokine levels in T2DM rats. (**A**) TNF-α, (**B**) IL-1β, (**C**) IL-6, and (**D**) IFN-γ levels in serum of different groups, n = 6, * *p* < 0.05, ** *p* < 0.01, *** *p* < 0.001.

Chronic low-grade inflammation is a hallmark of T2DM and its associated complications [42,43]. Analysis of serum inflammatory cytokines revealed that diabetic rats in the model group exhibited significantly elevated levels of TNF-α, IL-1β, IL-6, and IFN-γ (Figure 9, Table S13), indicating the presence of pronounced systemic inflammation. SIP treatment moderately reduced the levels of these cytokines, whereas SIP@CS-TPP treatment produced a much stronger inhibitory effect, with cytokine levels markedly lower than those in both the model and free SIP groups and approaching those of the positive control. Collectively, these findings indicate that SIP@CS-TPP effectively attenuates diabetes-associated inflammatory responses, thereby synergistically improving dysregulated glucose and lipid metabolism as well as insulin resistance.

## 4. Discussion

Natural polysaccharides have attracted considerable attention as adjunctive interventions for T2DM due to their favorable biocompatibility and multi-target metabolic regulatory potential [44,45]. However, the therapeutic efficacy of polysaccharides in vivo is often severely limited by poor oral stability, low intestinal permeability, and restricted bioavailability. Addressing these delivery-related barriers remains a major challenge for translating polysaccharide-based bioactives into effective oral interventions. In this study, we focused on improving oral delivery efficiency and developed a CS-TPP-based nanodelivery system to enhance the in vivo performance of SIP, systematically evaluating its delivery advantages and therapeutic potential in a T2DM model.

Through comprehensive formulation optimization and physicochemical characterization, SIP@CS-TPP nanoparticles with uniform particle size, positive surface charge, freeze-drying feasibility, and good long-term stability were successfully constructed. In vitro release studies demonstrated that this nanocarrier effectively suppressed rapid SIP release under simulated gastric conditions, while enabling sustained and controllable release in intestinal environments. Kinetic analysis indicated that SIP release was predominantly governed by diffusion-controlled mechanisms. These findings suggest that the CS-TPP nanostructure provides effective protection for SIP during gastrointestinal transit, thereby creating favorable conditions for its intestinal bioactivity.

Ex vivo intestinal permeation experiments showed that the CS-TPP nanodelivery system enhanced the trans-intestinal transport of SIP, with the Papp increased by approximately 3.7-fold compared with free SIP. In this study, the paracellular pathway was the dominant mechanism for the enhanced SIP permeation. Transcellular uptake played an auxiliary synergistic role. The CS used in this study had a degree of deacetylation of ≥85% and a weight-average molecular weight of 100 kDa. CS in this deacetylation range contains abundant free cationic amino groups [46]. These groups can reversibly interact with negatively charged components on the intestinal mucosal surface, as well as epithelial tight junction-related proteins (occludin and ZO-1) [47,48]. This interaction transiently opens intercellular tight junctions and widens the paracellular transport pathway, which is the core driver of the increased Papp. Meanwhile, the strong mucoadhesive property conferred by the cationic characteristic can prolong the intestinal retention time of nanoparticles. It further promotes the transcellular endocytosis of nanoparticles and synergistically enhances the transmembrane transport of SIP [49]. The above results confirm that the CS-TPP nanocarrier can effectively improve the intestinal transmembrane transport behavior of SIP. They also provide experimental evidence to explain the enhanced in vivo efficacy of SIP.

In vivo pharmacodynamic evaluations revealed that SIP@CS-TPP exhibited clear advantages in improving the overall metabolic status of diabetic animals. Compared with free SIP, the nanoparticle formulation achieved more pronounced effects in reducing fasting blood glucose, improving glucose tolerance, alleviating insulin resistance, and normalizing dyslipidemia. Although CS itself has been reported to exert mild metabolic regulatory effects [50], the consistently superior performance of SIP@CS-TPP relative to free SIP at the same dose strongly indicates that enhanced delivery efficiency, rather than intrinsic material effects or dose escalation, is the primary contributor to the observed therapeutic benefits.

Previous studies investigating the antidiabetic potential of natural polysaccharides have predominantly emphasized their antioxidant or anti-inflammatory properties [51,52], often overlooking the critical constraints imposed by in vivo delivery. This has resulted in a substantial gap between promising in vitro activity and modest in vivo efficacy. In contrast, the present study adopts a delivery-oriented strategy, demonstrating that improving gastrointestinal stability and intestinal permeability can systematically amplify the metabolic regulatory effects of SIP. This “delivery amplification” paradigm provides a more broadly applicable solution for advancing the oral use of polysaccharide-based therapeutics. At the mechanistic level, SIP@CS-TPP significantly attenuated oxidative stress and reduced the levels of multiple pro-inflammatory cytokines in diabetic animals. Accumulating evidence indicates that these inflammatory factors play a pivotal role in insulin resistance and diabetes-associated inflammation [53,54,55]. Our findings suggest that enhanced delivery enables SIP to more effectively modulate this inflammatory axis, thereby synergistically improving glucose-lipid metabolic disturbances and insulin sensitivity.

Several limitations of this study should be acknowledged. First, there are limitations in the structural characterization of the nanocarrier. In this study, FTIR was only used as an auxiliary technique to indicate the potential ionic interaction between CS and TPP, combined with indirect evidence such as particle size and zeta potential to support the formation of cross-linked nanoparticles. However, more direct characterization techniques were not used to provide definitive structural evidence for the cross-linking, which is a limitation of this study. In addition, the improved permeation of SIP after encapsulation may be attributed to the combined effects of enhanced mucoadhesion and modulation of paracellular transport, but the specific molecular mechanism has not been directly verified. Meanwhile, the long-term in vivo safety profile of the nanoparticle system was not systematically evaluated, and potential chronic toxicological effects require further investigation. Second, although enhanced delivery was associated with enhanced metabolic efficacy, the relative contribution of the nanocarrier itself was not independently assessed, as a blank CS-TPP control group was not included in the in vivo experiments. Third, the impact of SIP@CS-TPP on gut microbiota composition and function was not explored. Given the important role of the gut microbiota in mediating polysaccharide-induced metabolic regulation, future studies should further elucidate the contribution of the gut microbiota-metabolism axis to the observed therapeutic effects.

## 5. Conclusions

In this study, a CS-TPP oral nanodelivery system was developed to improve the metabolic regulatory effects of SIP in type 2 diabetes. SIP@CS-TPP nanoparticles exhibited favorable physicochemical properties, freeze-drying feasibility, and long-term stability. They effectively protected SIP under simulated gastrointestinal conditions and achieved pH-responsive release via a diffusion-dominated mechanism. Ex vivo experiments further confirmed that this system significantly enhanced the intestinal transmembrane transport efficiency of SIP. In a type 2 diabetic animal model, SIP@CS-TPP remarkably improved glycemic control, glucose tolerance, insulin resistance, dyslipidemia, oxidative stress, and inflammatory status compared with free SIP, demonstrating that optimized oral delivery can effectively enhance the in vivo metabolic regulatory efficacy of SIP. This study provides a feasible strategy for the oral delivery modification of natural polysaccharide-based bioactive agents. It also offers an experimental basis and translational reference for the application of SIP in metabolic diseases, which is of great significance for advancing preclinical research of polysaccharide formulations in diabetes and related complications. In the future, we will conduct more systematic research on long-term safety, tissue distribution, and the gut microbiota–metabolism axis, to provide a more comprehensive scientific basis for the subsequent clinical translation of this delivery system.

## Figures and Tables

**Table 1:** Mathematical models of the regression for in vitro release profiles of SIP@CS-TPP.

	Function	Equation	R^2^
pH 1.2	Zero order	Q = 5.12t + 0.83	0.887
	First order	ln(100 − Q) = 4.60 − 0.052t	0.821
	Higuchi	Q = 11.8t^1/2^ − 1.21	0.954
	Peppas–Sahlin	Q = 4.12t^0.45^ + 0.87t^0.90^	0.948
	Korsmeyer–Peppas	Q = 8.7t^0.88^	0.918
pH 6.8	Zero order	Q = 5.31t + 2.78	0.952
	First order	ln(100 − Q) = 4.58 − 0.085t	0.931
	Higuchi	Q = 17.2t^1/2^ − 1.25	0.987
	Peppas–Sahlin	Q = 8.95t^0.45^ + 1.52t^0.90^	0.985
	Korsmeyer–Peppas	Q = 9.49t^0.72^	0.975
pH 7.4	Zero order	Q = 8.52t + 11.84	0.831
	First order	ln(100 − Q) = 4.59 − 0.202t	0.967
	Higuchi	Q = 28.4t^1/2^ − 1.78	0.992
	Peppas–Sahlin	Q = 22.36t^0.45^ + 3.48t^0.90^	0.991
	Korsmeyer–Peppas	Q = 17.6t^0.79^	0.986

## Data Availability

The data that support the findings of this study are available in the manuscript. Extra data used to support this study are available from the corresponding author upon request.

## Supplementary Materials

The following supporting information can be downloaded at: https://www.mdpi.com/article/10.3390/pharmaceutics18050561/s1, Table S1: Optimization of formulation parameters for CS/TPP nanoparticles, n = 3; Table S2: Encapsulation efficiency and drug loading of SIP at different initial SIP concentrations, n = 3; Table S3: Changes in particle size and PDI of freeze-dried SIP@CS-TPP nanoparticles during storage at room temperature for up to 6 months, n = 3; Table S4: Cumulative release profiles of SIP@CS-TPP nanoparticles under pH 1.2, n = 3; Table S5: Cumulative release profiles of SIP@CS-TPP nanoparticles under pH 6.8 and 7.4, n = 3; Table S6: Simulated gastrointestinal sequential release profile of SIP@CS-TPP nanoparticles, n = 3; Table S7: Cumulative permeation of free SIP and SIP@CS-TPP across isolated rat small intestine over 6 h, n = 3; Table S8: Papp of SIP and SIP@CS-TPP, n = 3; Table S9: Changes in body weight during the 8-week treatment period, n = 6; Table S10: FBG levels during the treatment period, n = 6; Table S11: FBG levels at week 8, n = 6; Table S12: Effects of SIP@CS-TPP on oral glucose tolerance in T2DM rats. Blood glucose levels measured during OGTT at 0, 30, 60, and 90 min in control, model, SIP, SIP@CS-TPP, and positive control groups, n = 6; Table S13: Effects of SIP@CS-TPP on serum lipid metabolism (TC, TG, LDL-C, and HDL-C), oxidative stress (SOD, GSH, CAT, and MDA), and inflammatory cytokine (TNF-α, IL-1β, IL-6, and IFN-γ) in T2DM rats, n = 6.

## Abbreviations

The following abbreviations are used in this manuscript:

CAT	Catalase
CS	Chitosan
CS-TPP	Chitosan/tripolyphosphate
DAPI	4′,6-diamidino-2-phenylindole
DLS	Dynamic light scattering
FBG	Fasting blood glucose
FINS	Fasting insulin
FTIR	Fourier transform infrared spectroscopy
GSH	Glutathione
HDL-C	High-density lipoprotein cholesterol
HOMA-IR	Homeostasis model assessment of insulin resistance
IFN-γ	Interferon gamma
IL-1β	Interleukin-1 beta
IL-6	Interleukin-6
IR820	Near-infrared fluorescent dye IR820
LDL-C	Low-density lipoprotein cholesterol
MDA	Malondialdehyde
OGTT	Oral glucose tolerance test
Papp	Apparent permeability coefficient
PBS	Phosphate-buffered saline
PDI	Polydispersity index
P-IκB-α	Phosphorylated inhibitor of nuclear factor kappa B alpha
P-P65	Phosphorylated NF-κB p65
SD	Sprague–Dawley
SIP	Snow lotus polysaccharide
SIP@CS-TPP	Snow lotus polysaccharide-loaded chitosan/tripolyphosphate nanoparticles
SOD	Superoxide dismutase
STZ	Streptozotocin
TC	Total cholesterol
TEM	Transmission electron microscopy
TG	Triglycerides
TLR4	Toll-like receptor 4
TPP	Sodium tripolyphosphate
T2DM	Type 2 diabetes mellitus
