# Supplementary material for: Oral Chitosan–Tripolyphosphate Nanoparticles Enhance the Metabolic Regulatory Effects of Snow Lotus Polysaccharide in Type 2 Diabetes
Source: Pharmaceutics. 2026 Apr 30;18(5):561. doi: 10.3390/pharmaceutics18050561 (PMC13210455; doi:10.3390/pharmaceutics18050561)
Supplement: Supplementary file 1 [file pharmaceutics-18-00561-s001.zip › pharmaceutics-4224799-supplementary.pdf]

Table S1 Optimization of formulation parameters for CS/TPP nanoparticles, n = 3

	Condition	Size (nm)	PDI
Stirring time (s)	0.25	442.1 ± 22.5	0.58 ± 0.21
	0.50	408.4 ± 23.8	0.29 ± 0.15
	1.00	384.8 ± 19.4	0.30 ± 0.12
	1.50	359.8 ± 16.4	0.25 ± 0.15
	2.00	298.1 ± 15.2	0.19 ± 0.06
Molecular weight of CS	0.25	327.8 ± 15.9	0.25 ± 0.05
	0.50	298.4 ± 14.3	0.18 ± 0.04
	1.00	276.6 ± 19.4	0.15 ± 0.06
	1.50	267.4 ± 13.2	0.19 ± 0.03
pH	3.0	245.2 ± 13.5	0.22 ± 0.06
	3.5	214.4 ± 16.4	0.22 ± 0.07
	4.0	192.1 ± 12.5	0.16 ± 0.03
	4.5	185.3 ± 11.4	0.15 ± 0.02
	5.0	167.4 ± 10.2	0.11 ± 0.02
	5.5	193.5 ± 17.8	0.16 ± 0.06
	6.0	246.1 ± 19.2	0.18 ± 0.09
	6.5	351.8 ± 20.5	0.26 ± 0.11
CS concentration (mg/mL)	0.50	264.2 ± 16.7	0.18 ± 0.03
	0.75	225.9 ±15.8	0.17 ± 0.04
	1.00	194.2 ±21.8	0.15 ± 0.03
	1.50	219.5 ±22.5	0.21 ± 0.05
	2.00	245.9 ± 24.8	0.15 ± 0.03
TPP concentration (mg/mL)	0.5	124.5 ± 8.91	0.12 ± 0.04
	0.75	177.2 ± 13.5	0.15 ± 0.05
	1	189.1 ± 15.3	0.16 ± 0.04
	1.25	195.7 ± 16.4	0.11 ± 0.06
	1.5	244.8 ± 21.2	0.17 ± 0.06
CS: TPP mass ratio	3:1	269.2 ± 25.9	0.11 ± 0.05
	4:1	224.3 ± 16.2	0.15 ± 0.06
	5:1	169.2 ± 16.4	0.09 ± 0.03
	6:1	181.2 ± 15.7	0.16 ± 0.06
	7:1	244.8 ± 13.8	0.18 ± 0.04

Table S2 Encapsulation efficiency and drug loading of SIP at different initial SIP concentrations, n = 3

Dosage (mg)	EE (%)	DL (%)
1	73.21 ± 4.73%	37.90 ± 1.52%
2	65.07 ± 3.87%	35.16 ± 1.35%
3	61.23 ± 4.86%	33.79 ± 1.78%
4	49.83 ± 5.22%	29.34 ± 2.17%
5	34.75 ± 3.75%	22.46 ± 1.88%

Table S3 Changes in particle size and PDI of freeze-dried SIP@CS-TPP nanoparticles during storage at room temperature for up to 6 months, n = 3

Storage time (month)	Size (nm)	PDI
1	197.54 ± 15.64	0.17 ± 0.04
2	203.53 ± 14.32	0.22 ± 0.05
3	192.64 ± 17.64	0.16 ± 0.04
4	199.61 ± 12.75	0.17 ± 0.03
5	204.39 ± 19.36	0.19 ± 0.05
6	201.24 ± 16.32	0.16 ± 0.06

Table S4 Cumulative release profiles of SIP@CS-TPP nanoparticles under pH 1.2, n = 3

Time (h)	0	0.25	0.5	1	1.5	2	2.5	3	4
pH1.2	0	2.87 ±0.64%	6.25 ± 0.92%	11.93 ± 0.94%	13.86 ± 1.25%	14.43 ± 1.42%	19.04 ± 1.31%	20.54 ± 1.54%	21.38 ± 1.62%

Table S5 Cumulative release profiles of SIP@CS-TPP nanoparticles under pH 6.8 and 7.4, n = 3

Time (h)	0	0.5	1	2	3	4	6	8	10
pH 6.8	0	6.32 ±0.53%	12.75 ± 0.73%	20.54 ± 1.22%	29.91 ± 1.68%	36.85 ± 2.53%	46.32 ± 3.71%	50.17 ± 4.02%	54.61 ± 4.93%
pH 7.4	0	19.63 ± 1.25%	32.14 ± 2.98%	46.75 ± 3.46%	57.43 ± 4.98%	69.75 ± 5.64%	80.32 ± 6.53%	84.43 ± 6.87%	87.21 ± 6.12%

Table S6 Simulated gastrointestinal sequential release profile of SIP@CS-TPP nanoparticles, n = 3

Time	0	0.5	1	2	3	4	5	6	7	8	9	10	12
	pH 1.2				pH 6.8				pH 7.4				
Release (%)	0	5.69 ± 0.43%	9.97 ± 0.78%	14.26 ± 1.26%	22.84 ± 1.95%	29.41 ± 2.53%	35.85 ± 3.01%	41.63 ± 3.86%	55.73 ± 4.93%	67.43 ± 5.63%	78.31 ± 6.32%	87.43 ± 7.98%	90.41 ± 8.01%

Table S7 Cumulative permeation of free SIP and SIP@CS-TPP across isolated rat small intestine over 6 h, n = 3

Time (h)	SIP	SIP@CS-TPP
0	0	0
0.5	1.53 ± 0.16%	5.33 ± 0.37%
1	2.32 ± 0.21%	12.75 ± 0.94%
2	6.32 ± 0.47%	19.86 ± 1.65%
3	9.41 ± 0.78%	27.64 ± 2.64%
4	11.24 ± 0.92%	35.85 ± 3.19%
5	13.76 ± 0.99%	46.65 ± 4.28%
6	16.33 ± 1.25%	59.83 ± 5.31%

Table S8 Papp of SIP and SIP@CS-TPP, n = 3

	SIP×10^{-8}(cm/s)	SIP@CS-TPP×10^{-8}(cm/s)
1	9.630809153	35.28544468
2	10.36801132	38.4170795
3	8.893606983	33.71962727

Table S9 Changes in body weight during the 8-week treatment period, n = 6

Control	Model	SIP	SIP@CS-TPP	Positive
315.21 ± 3.38	338.72 ± 6.42	330.28 ± 6.28	340.96 ± 7.62	335.68 ± 6.28
328.89 ± 4.64	360.64 ± 4.39	350.24 ± 5.76	356.86 ± 6.84	358.82 ± 5.37
352.67 ±3.74	363.26 ± 5.64	362.67 ± 5.85	370.69 ± 7.06	368.96 ±5.85
383.64 ±4.82	348.89 ± 4.78	359.72 ± 4.84	368.86 ± 6.23	356.74 ± 6.02
401.85 ± 5.26	302.59 ± 4.62	328.22 ± 5.68	345.18 ± 4.98	329.47 ± 6.82

Table S10 FBG levels during the treatment period, n = 6

Control	Model	SIP	SIP@CS-TPP	Positive
5.91 ± 0.63	19.85 ± 0.74	19.73 ± 0.64	19.43 ± 0.84	19.53 ± 0.64
5.7 ± 0.84	21.33 ± 0.68	19.34 ± 0.82	18.62 ± 0.65	17.92 ± 0.82
5.23 ± 0.76	22.82 ± 0.80	17.42 ± 0.78	16.64 ± 0.89	16.28 ± 0.58
5.44 ± 0.79	24.63 ± 0.78	14.9 ± 0.69	13.66 ± 0.74	13.91 ± 0.78
5.56 ± 0.60	25.79 ± 1.59	13.51 ± 1.31	12.87 ± 1.15	11.96 ± 0.93

Table S11 FBG levels at week 8, n = 6

	Control	Model	SIP	SIP@CS-TPP	Positive
FINS	14.21 ± 0.46	30.24 ± 1.34	26.02 ± 0.98	21.3 ± 1.02	18.46 ± 0.96
HOMA-IR	3.54 ± 0.21	34.41 ± 2.04	18.07 ± 1.03	12.59 ± 0.96	13.76 ± 0.91

Table S12 Effects of SIP@CS-TPP on oral glucose tolerance in T2DM rats. Blood glucose levels measured during OGTT at 0, 30, 60, and 90 min in control, model, SIP, SIP@CS-TPP, and positive control groups, n = 6

Time (min)	Control	Model	SIP	SIP@CS-TPP	Positive
0	7.65 ± 0.63	20.23 ± 1.49	19.95 ± 1.75	19.68 ± 1.20	19.11 ± 1.23
30	11.72 ± 0.94	38.38 ±1.94	30.33 ± 2.06	27.27 ± 2.07	25.60 ± 1.83
60	10.27 ± 0.90	33.65 ± 2.06	26.37 ± 1.87	20.76 ± 1.69	19.32 ± 1.42
90	9.16 ± 0.78	30.54 ± 2.09	21.69 ± 1.57	18.81 ± 1.65	16.16 ± 1.28

Table S13 Effects of SIP@CS-TPP on serum lipid metabolism (TC, TG, LDL-C, and HDL-C), oxidative stress (SOD, GSH, CAT, and MDA), and inflammatory cytokine (TNF-α, IL-1β, IL-6, and IFN-γ) in T2DM rats, n = 6

	Control	Model	SIP	SIP@CS-TPP	Positive
TC	1.54 ± 0.10	3.85 ± 0.31	3.05 ± 0.21	2.59 ± 0.19	1.95 ± 0.15
TG	0.57 ± 0.03	1.23 ± 0.07	1.06 ± 0.06	0.82 ± 0.05	0.84 ± 0.04
LDL-C	0.82 ± 0.02	2.15 ± 0.10	1.65 ± 0.05	1.22 ± 0.04	1.52 ± 0.05
HDL-C	1.42 ± 0.05	0.92 ± 0.03	0.98 ± 0.03	1.25 ± 0.05	1.36 ± 0.05
SOD	585.6 ± 30.02	289.3 ± 10.24	293.5 ± 11.03	496.8 ± 19.98	487.6 ± 22.12
GSH	208.6 ± 10.03	109.5 ± 5.12	115.6 ± 5.96	181.3 ± 9.11	176.4 ± 8.99
CAT	78.6 ± 6.02	45.5 ± 2.98	50.2 ± 2.96	58.4 ± 4.06	72.3 ± 6.09
MDA	4.7 ± 0.3	15.2 ± 1.2	10.8 ± 0.6	6.8 ± 0.4	7.3 ± 0.6
TNF-α	0.15 ± 0.01	3.23 ± 0.21	1.74 ± 0.13	0.88 ± 0.4	0.54 ± 0.02
IL-1β	9.2 ± 0.4	291.3 ± 15.1	157.4 ± 5.0	87.8 ± 3.9	108.5 ± 4.9
IL-6	0.16 ± 0.01	1.58 ± 0.10	1.14 ± 0.08	0.59 ± 0.04	0.69 ± 0.05
IFN-γ	0.21 ± 0.01	1.92 ± 0.12	1.07 ± 0.07	0.68 ± 0.05	0.38 ± 0.02
